# Genomic-Enabled Prediction of Ordinal Data with Bayesian Logistic Ordinal Regression

**DOI:** 10.1534/g3.115.021154

**Published:** 2015-08-18

**Authors:** Osval A. Montesinos-López, Abelardo Montesinos-López, José Crossa, Juan Burgueño, Kent Eskridge

**Affiliations:** * Facultad de Telemática, Universidad de Colima, C.P. 28040 Colima, Colima, México; † Departamento de Estadística, Centro de Investigación en Matemáticas (CIMAT), 36240 Guanajuato, México; ‡ Biometrics and Statistics Unit, International Maize and Wheat Improvement Center (CIMMYT), 06600 México, Distrito Federal, México; § University of Nebraska, Statistics Department, Lincoln, Nebraska 68583-0963

**Keywords:** Bayesian ordinal regression, genomic selection, probit, logit, Gibbs sampler, GenPred, shared data resource

## Abstract

Most genomic-enabled prediction models developed so far assume that the response variable is continuous and normally distributed. The exception is the probit model, developed for ordered categorical phenotypes. In statistical applications, because of the easy implementation of the Bayesian probit ordinal regression (BPOR) model, Bayesian logistic ordinal regression (BLOR) is implemented rarely in the context of genomic-enabled prediction [sample size (*n*) is much smaller than the number of parameters (*p*)]. For this reason, in this paper we propose a BLOR model using the Pólya-Gamma data augmentation approach that produces a Gibbs sampler with similar full conditional distributions of the BPOR model and with the advantage that the BPOR model is a particular case of the BLOR model. We evaluated the proposed model by using simulation and two real data sets. Results indicate that our BLOR model is a good alternative for analyzing ordinal data in the context of genomic-enabled prediction with the probit or logit link.

Genomic-enabled prediction models are revolutionizing animal and plant breeding. There is some evidence that they are powerful for predicting the genomic merit of animals and plants based on high-density single-nucleotide polymorphism (SNP) marker panels and are being recommended increasingly for genomic prediction in human health (Yang and Tempelman 2012). However, most genomic-enabled prediction models assume a continuous and normally distributed phenotype. Because often this assumption is not fulfilled, researchers normally approach phenotypes in three ways: (a) they ignore the lack of normality in the phenotypes; (b) they transform the non-normal phenotype to approximate it to normality; or (c) they use generalized linear mixed models (GLMMs) to model the appropriate distribution of the phenotype (Stroup 2015).

The use of the first approach is justified for large sample sizes with the central limit theorem, which states that treatment means have an approximate normal distribution if the sample size is large enough. However, there is a lot of evidence indicating that the first approach produces highly biased results for small- and moderate-sample sizes (Stroup 2012, 2015). Transformations introduced by Bartlett (1947) for non-normal data were proposed for variance-stabilization to fulfill the assumption of homogeneous variance; they are still considered standard procedures in many agricultural disciplines. Implementation with transformations is equal to that for phenotypes normally distributed (based on the linear model). However, there is mounting evidence that transformations do more harm than good for the models required by most agricultural research, because the use of the linear model with or without transformed data produces a great loss of accuracy and power (Stroup 2015), mostly in small sample sizes.

Nelder and Wedderburn (1972) introduced generalized linear models, a major departure from the usual approach to non-normal data. GLMMs extend the linear model theory to accommodate non-normal data with heterogeneous variance and even correlated observations. Viewed through the GLMM lens, the pre-1990s understanding of non-normal data––still pervasive in the agricultural research community––is antiquated at best, obsolete at worst. Today, small sample investigations are providing an increasing body of evidence that GLMMs work as well in practice as they do in theory because they produce more accuracy and power than approaches (a) and (b). Also, now there are textbooks and software available for the implementation of GLMMs, although the implementation of approaches (a) and (b) is still dominant in agricultural research (Stroup 2015). In genomic-enabled prediction, the use of GLMMs is still in its early stages because their implementation is not straightforward given that the number of observations (*n*) is usually smaller than the number of covariates (*p*) included in the model. In addition, a complex dependence structure is observed among covariates (markers) and observations (lines) due to the joint involvement of biological processes and pathways.

To overcome this situation in the pregenomic era, Gianola (1980, 1982) and Gianola and Foulley (1983) proposed a probit (threshold) model for dealing with ordinal categorical traits in animal breeding. This probit model was extended to deal with *p >> n* in the genomic era by González-Recio and Forni (2011) and Villanueva *et al.* (2011) for binary trials, and by Wang *et al.* (2012) and Montesinos-López *et al.* (2015) for more than two ordinal categories. Also, BGLR (Bayesian generalized linear regression), software developed for genomic-enabled prediction that is able to deal with normal, binary, ordinal, and censored data (de los Campos and Perez-Rodriguez 2013; Perez-Rodriguez and de los Campos 2014), is now available. However, no GLMMs are available for genomic-enabled prediction for counts and percentage phenotypes.

For modeling ordinal categorical phenotypes, the ordinal logistic regression model is often preferred over the ordinal probit model in statistical applications, because it provides regression coefficients that are more interpretable due to their connection to odds ratios (Zucknick and Richardson 2014). However, in genomic-enabled prediction (when *p >> n*), only the Bayesian probit model is frequently implemented, given that Bayesian methods that introduce sparseness through additional priors on the model size are very well-suited to this problem. Therefore, because of the lack of a Bayesian logistic ordinal model analogous to the Bayesian probit ordinal model that uses a data augmentation approach, the logistic model is not practical for genomic selection.

Both logistic and normal distributions are symmetric with a basic, unimodal “bell curve” shape. The only difference is that the logistic distribution has a somewhat heavier tails, which means that it is less sensitive to outlying data (and hence somewhat more robust for modeling mis-specifications or erroneous data). This is another advantage of logistic regression over probit regression. Because of its easy implementation, the use of BPOR is extremely common, even though it is less robust for modeling mis-specifications and its coefficients are less interpretable. Because of the aforementioned properties of the logistic model, some researchers have proposed approximations to logit regression. For example, Bartholomew and Knott (1999) proposed $logit\left(u\right)=k\times {\text{Φ}}^{-1}(u)$, where Φ is the cumulative density function for the standard normal distribution and k=1.814, whereas Camilli (1994) proposed using *k* = 1.702, obtained by minimizing the maximum distance between two cumulative distribution functions. Although Amemiya (1981) proposed a value of *k* = 1.6 and computed tables for representative values of the density function for different values of *k*, he did not explain why he used k=1.6. More recently, Savalei (2006) obtained a value of *k* = 1.75 based on minimizing the Kullback-Leibler information. However, although some of these approximations do a reasonable job of approximating the logistic distribution, they are only approximations, and it goes without saying that an exact solution is preferred.

In this paper, we propose a Bayesian logistic ordinal regression (BLOR) model for genomic-enabled prediction by using a data augmentation approach. We illustrate our proposed method with simulation and real data. We compare the BLOR with the Bayesian probit ordinal regression (BPOR) model with and without approximation.

## Materials and Methods

### Gray leaf spot (GLS) and Septoria data sets

GLS is one of the most important foliar diseases of maize worldwide. The GLS data set is composed of 278 maize lines; the ordinal trait measured in each line was GLS [1 (no disease), 2 (low infection), 3 (moderate infection), 4 (high infection), 5 (complete infection)] caused by the fungus *Cercospora zeae-maydis*, evaluated in three environments (México, Zimbabwe, and Colombia). These data are part of a data set previously analyzed by Crossa *et al.* (2011), González-Camacho *et al.* (2012), and Montesinos-López *et al.* (2015). Genotypes of all 278 lines were obtained using the 55k SNP Illumina platform. SNPs with >10% missing values or a minor allele frequency of ≤ 0.05 were excluded from the data. After line-specific quality control (applying the same quality control to each line separately), the maize data still contained 46,347 SNPs.

On the other hand, the Septoria data set contains 268 wheat lines planted in Toluca, México, in 2010, and the trait (Septoria scores) was measured using an ordinal four-point scale. Genotypes of these lines were obtained with 45,000 genotype by sequencing (GBS), following the protocol of Poland *et al.* (2012). We kept only 13,913 GBS that had <50% missing data; after filtering for minor allele frequency, we ended up with 6787 GBS that were used in the analysis.

For the implementation of the proposed model, we formed five data sets from these two real data sets (GLS and Septoria), four from the GLS data set and one from the Septoria data set. The first three data sets formed from GLS correspond to each environment in which they were evaluated for GLS; the last one was formed by pooling the data from the three environments (information from the three environments without taking into account the environments as covariates).

### Bayesian logistic ordinal regression

Let $y=\left\{y_{ij}\right\}$ ($i=1,\;\ldots ,I;\;j=1,2,\ldots ,n_{i}),\;\;$where *i* represents the genotype and *j* denotes the number of replicates or experimental units of each genotype. The total number of observations is $n=\overset{I}{\underset{i=1}{\sum }}n_{i}.\;\;$In other words, the observed vector $y_{i}$ contains $n_{i}$ elements, and the *n*-dimensional vector **y** of all responses can be written as $y^{T}=\left(y_{1}^{T},y_{2}^{T},\ldots ,y_{I}^{T}\right)$. The response variable $y_{ij}$ represents an assignment into one of *C* mutually exclusive and exhaustive categories that follow an order. Therefore, the ordinal logistic regression model can be written in terms of a latent response variable $l_{ij}$ as follows:

$$l_{ij}=x_{\mathrm{ij}}^{T}\beta +b_{i}+\varepsilon_{ij}$$ (1)

where $l_{ij}$ are called “liabilities”, $\varepsilon_{ij}∼L\left(0,1\right)$, where L(.) denotes the logistic distribution, and the vectors $x_{ij}$ (*p* × 1) are explanatory variables associated with the fixed effects ***β***. The random effect $b_{i}∼N(0,\sigma_{b}^{2})$. In genomic-enabled prediction, $b={\left(b_{1},\ldots ,b_{I}\right)}^{T}∼N\left(0,G\sigma_{b}^{2}\right).\;\;$ Since $l_{ij}$ are unobservable and can be measured indirectly by an observable ordinal variable $y_{ij}$, then $l_{ij}$ can be defined by:

$$y_{ij}=\{\begin{array}{c} 1\;if\;-\infty <l_{ij}<\gamma_{1},\\ 2\;if\;\gamma_{1}<l_{ij}<\gamma_{2},\\ \vdots \\ C\;if\;\gamma_{C-1}<l_{ij}<\infty \end{array}$$

This means that $l_{ij}$ is divided by thresholds into *C* intervals, corresponding to *C* ordered categories. The first threshold, $\gamma_{1}$, defines the upper bound of the interval corresponding to observed outcome 1. Similarly, threshold $\gamma_{C-1}$ defines the lower bound of the interval corresponding to observed outcome *C*. Threshold $\gamma_{c}$ defines the boundary between the interval corresponding to observed outcomes *c* − 1 and *c* for (*c* = 1,2,…, *C* − 1). Threshold parameters are $\gamma^{T}=\left(\gamma_{min}<\gamma_{1}<\cdots <\gamma_{C-1}<\gamma_{max}\right)$ with $\gamma_{min}=-\infty$, and $\gamma_{max}=\infty .\;$

Assuming that the error term $\varepsilon_{ij}$ of the latent response $l_{ij}$ is distributed as L(0,1), the cumulative response probability for the *c* category of the ordinal outcome $y_{ij}$ is:

$$P(y_{ij}\leq c|\beta ,b)=\pi_{ij\left(c\right)}=P(l_{ij}\leq \gamma_{c}|\beta ,b)=\text{P}\left(x_{ij}^{T}\beta +b_{i}+\varepsilon_{ij}\leq \gamma_{c}\right)$$

$$=\text{P}\left(\varepsilon_{ij}\leq \gamma_{c}-x_{ij}^{T}\beta -b_{i}\right),\;$$

for  c=1,2,…,C−1.

$$=\frac{\text{exp}(\gamma_{c}-x_{ij}^{T}\beta -b_{i})}{1+\text{exp}(\gamma_{c}-x_{ij}^{T}\beta -b_{i})}\;$$ (2)

Similarly, model (2) can be written as a cumulative logit model:

$$log\left(\frac{\pi_{ij\left(c\right)}}{1-\pi_{ij\left(c\right)}}\right)=\gamma_{c}-x_{ij}^{T}\beta -b_{i},\;\text{for}\;c=1,2,\ldots ,C-1.$$

This GLMM model is described by: (1) two distributions, one for observations in the response variable $(y_{ij(1)},\;y_{ij(2)},\ldots ,y_{ij(C)}|\beta ,b)∼$Multinomial $(1,\;\pi_{ij(1)},\;\pi_{ij(2)},\ldots ,\pi_{ij(C)}$), where ***β*** is the p × 1 vector of fixed effects, and another for the random effects, $b_{i}∼N(0,\sigma_{b}^{2})$ or $b∼N\left(0,G\sigma_{b}^{2}\right)$, where *b_i_* is the effect of line *i*; (2) linear predictor $\eta_{ij(c)}=\gamma_{c}-x_{ij}^{T}\beta -b_{i}$, where $\eta_{ij(c)}$ denotes the *c*^th^ link (*c* = 1,2,…, *C* − 1) for the fixed and random effects combination, $\gamma_{c}$ is the intercept (threshold) for the *c*^th^ link, and $x_{ij}^{T}$ are known row incidence vectors corresponding to fixed effects in ***β***. Because there are *C* categories, a total of *C* − 1 link functions are required to fully specify the model; and (3) link function: cumulative logit {$\eta_{ij(c)}=log\left(\frac{\pi_{ij\left(c\right)}}{1-\pi_{ij\left(c\right)}}\right)$, (*c* = 1,2,…, *C* − 1)}.

Using the inverse link for this model, we can calculate $P(y_{ij}=c|\beta ,\;b)=\pi_{ij\left(c\right)}\;$as follows:

$$\pi_{ij\left(c\right)}=P\left(\gamma_{c-1}<l_{ij}<\gamma_{c}\right)=\frac{\text{exp}(\gamma_{c}-x_{\mathrm{ij}}^{T}\beta -b_{i})}{1+\text{exp}(\gamma_{c}-x_{ij}^{T}\beta -b_{i})}-\frac{\text{exp}(\gamma_{c-1}-x_{ij}^{T}\beta -b_{i})}{1+\text{exp}(\gamma_{c-1}-x_{ij}^{T}\beta -b_{i})}$$.

Since we have latent variables $l_{ij}$ distributed as $L\left(x_{ij}^{T}\beta +b_{i},1\right)$ and we observe $y_{ij}=c$ if, and only if, $\gamma_{c-1}<l_{ij}<\gamma_{c}$, then the joint posterior density of the parameter vector and latent variable becomes

$$P\left(\beta ,\gamma ,b,\sigma_{\beta }^{2},\sigma_{b}^{2},l|y\right)\propto P\left(y|l,\gamma \right)P\left(l|\beta ,b\right)P\left(\gamma \right)P(\beta |\sigma_{\beta }^{2})P\left(b|\sigma_{b}^{2}\right)P\left(\sigma_{\beta }^{2}\right)P\left(\sigma_{b}^{2}\right).$$

Let’s assume a scaled independent inverse chi-square $\chi^{-2}(\nu_{b},S_{b})$ prior for $\sigma_{b}^{2}$, a normal prior distribution for $\beta |\sigma_{\beta }^{2}∼N(\beta_{0},\Sigma_{0}\sigma_{\beta }^{2})$, a normal prior distribution for $b|\sigma_{b}^{2}∼N(0,G\sigma_{b}^{2})$, and also a $\chi^{-2}(\nu_{\beta },S_{\beta })$ prior for $\sigma_{\beta }^{2}$ (Gianola 2013). Following Sorensen *et al.* (1995), the prior for the *C* − 1 unknown thresholds has been given as order statistics from U($\gamma_{\text{min}}$, $\gamma_{\text{max}})$ distribution,

$$P\left(\gamma \right)=\left(C-1\right)!{\left(\frac{1}{\gamma_{\text{max}}-\gamma_{\text{min}}}\right)}^{C-1}I\left(\gamma \in T\right)$$

where $T=\{(\gamma_{1},\ldots ,\gamma_{\text{max}})|\gamma_{\text{min}}<\gamma_{1}<\cdots <\gamma_{C-1}<\gamma_{\text{max}}\}$.

The fully conditional posterior distributions are provided below and details of all derivations are given in Appendix A.

### Liabilities and Pólya-Gamma values

The fully conditional posterior distribution of liability $l_{ij}$ is a truncated normal distribution and its density is

$$P\left(l_{ij}|ELSE\right)=\frac{\varphi (x_{\mathrm{ij}}^{T}\beta +b_{i},1/\sqrt{\omega_{ij}})}{\text{Φ}\left(\gamma_{c}-x_{ij}^{T}\beta -b_{i}\right)-\text{Φ}\left(\gamma_{c-1}-x_{ij}^{T}\beta -b_{i}\right)}$$ (3)

For simplicity, *ELSE* is the data and the parameters, except the one in question. φ(.) is a normal density with parameters as indicated in the argument, Φ is the cumulative distribution function of a normal density with mean $x_{ij}^{T}\beta +b_{i}\;$and variance **$1/\sqrt{\omega_{ij}}$**, and the fully conditional posterior distribution of *ω_ij_* is

$$\omega_{ij}|ELSE∼PG\left(2,-l_{ij}+x_{ij}^{T}\beta +b_{i}\right)$$ (4)

### Regression coefficients (*β*)

The fully conditional posterior of ***β*** is as follows:

$$\beta |ELSE∼N_{p}\left({\tilde{\beta }}_{0},{\tilde{\Sigma }}_{0}\right)$$ (5)

where ${\tilde{\Sigma }}_{0}={\left(\Sigma_{0}^{-1}\sigma_{\beta }^{-2}+X^{T}D_{\omega }X\right)}^{-1}$, ${\tilde{\beta }}_{0}={\tilde{\Sigma }}_{0}(\Sigma_{0}^{-1}\sigma_{\beta }^{-2}\beta_{0}-X^{T}D_{\omega }Zb+X^{T}D_{\omega }l)$. With $l={\left[l_{1}^{T},\ldots ,\;l_{I}^{T}\right]}^{T}$, $l_{i}={\left[l_{i1}^{T},\ldots ,\;l_{in_{i}}^{T}\right]}^{T}$, **$X={\left[X_{1}^{T},\ldots ,\;X_{I}^{T}\right]}^{T}$**, $X_{i}={\left[x_{i1},\ldots ,\;x_{in_{i}}\right]}^{T}$, $Z=\left[\begin{array}{ccc} \begin{array}{cc} 1_{n_{1}} & 0\\ 0 & 1_{n_{2}} \end{array} & \begin{array}{c} \cdots \\ \cdots \end{array} & \begin{array}{c} 0\\ 0 \end{array}\\ \begin{array}{cc} \vdots & \vdots \end{array} & \ddots & \vdots \\ \begin{array}{cc} 0 & 0 \end{array} & \cdots & 1_{n_{I}} \end{array}\right]$, $D_{\omega }=diag\left(D_{\omega 1},..,D_{\omega I}\right)$, $D_{\omega i}=diag\left(\omega_{i1},\;\ldots ,\;\omega_{in_{i}}\right)$. It is important to point out that if we use a prior for β∝Constant (improper uniform distribution), then in ${\tilde{\Sigma }}_{0}$ and ${\tilde{\beta }}_{0}$ we need to make **0** the term $\Sigma_{0}^{-1}\sigma_{\beta }^{-2}$.

### Polygenic effects (*b*)

Now the fully conditional posterior of ***b*** is given as

$$b|ELSE∼N_{I}\left(\overset{˜}{b}=F\left(Z^{T}D_{\omega }l-Z^{T}D_{\omega }X\beta \right),F={\left(\sigma_{b}^{-2}G^{-1}+Z^{T}D_{\omega }Z\right)}^{-1}\right)\;$$ (6)

### Variance of polygenic effects

Next, the fully conditional posterior of $\sigma_{b}^{2}$ is

$$\sigma_{b}^{2}|ELSE∼\chi^{-2}({\tilde{\nu }}_{b}=\nu_{b}+I,{\tilde{S}}_{b}=(b^{T}G^{-1}b+\nu_{b}S_{b})/\nu_{b}+I)$$ (7)

### Threshold effects (***γ***)

The density of the fully conditional posterior distribution of the *c*^th^ threshold, $\gamma_{c}$, is

$$P\left(\gamma_{c}|ELSE\right)=\frac{1}{\min\left\{\min\;\left(l_{ij}|y_{ij}=c+1\right),\gamma_{c+1},\gamma_{\max\;\;}\right\}-\max\{\max\left(l_{ij}|y_{ij}=c\right),\gamma_{c-1},\gamma_{\min}\}}$$ (8)

### Variance of regression coefficients

The fully conditional posterior of $\sigma_{\beta }^{2}$ is

$$\sigma_{\beta }^{2}|ELSE∼\chi^{-2}({\tilde{\nu }}_{\beta }=\nu_{\beta }+p,{\tilde{S}}_{\beta }=[{\left(\beta -\beta_{0}\right)}^{T}\Sigma_{0}^{-1}(\beta -\beta_{0})+\nu_{\beta }S_{\beta }]/\nu_{\beta }+p)$$ (9)

### The Gibbs sampler

The Gibbs sampler is implemented by sampling repeatedly from the following loop:

1. Sample liabilities from the truncated normal distribution in (3).
2. Sample $\omega_{ij}$ values from the Pólya-Gamma distribution in (4).
3. Sample the regression coefficients from the normal distribution in (5).
4. Sample the polygenic effects from the normal distribution in (6).
5. Sample the variance effect ($\sigma_{b}^{2})$ from the scaled inverted χ^2^ distribution in (7).
6. Sample the thresholds from the uniform distribution in (8).
7. Sample the variance of regression coefficients ($\sigma_{\beta }^{2})$ from the scaled inverted χ^2^ distribution in (9).
8. Return to step 1 or terminate if chain length is adequate to meet convergence diagnostics.

In the absence of polygenic effects (**b**), the aforementioned Gibbs sampler can be used only by ignoring steps 4 and 5. If all marker effects are taken into account in the design matrix, ***X***, with a prior $\beta ∼N_{p}\left(0,\;I_{p}\sigma_{\beta }^{2}\right)$ for the beta regression coefficients, we end up with a threshold Bayesian ridge regression. This is a version for ordinal categorical data of the ridge estimator of Hoerl and Kennard (1970), since the posterior expectation of ***β*** is equal to $E\left(\beta |\;ELSE\right)={\left(X^{T}D_{\omega }X+I_{p}\sigma_{\beta }^{-2}\right)}^{-1}X^{T}D_{\omega }l$ with pseudo-response ***l***. Another important point is that by setting each $\omega_{ij}=1$, the aforementioned Gibbs sampler for the BLOR with the logistic link is reduced to the Gibbs sampler for the BPOR with the probit link proposed by Albert and Chib (1993). This implies that the proposed BLOR model is more general and includes the Gibbs sampler for the BPOR model as a particular case.

### Simulation study

The purpose of this simulation study is twofold: (1) to compare the performance of the proposed BLOR with: (a) the approximation resulting from using the estimates of the BPOR with $\log it\left(u\right)=1.75\times \Phi^{-1}\left(u\right)$, denoted as BLOR*, (b) with the results of using maximum likelihood estimators (MLEs) with logit link for ordinal data (MLLOR), and (c) the approximation resulting from multiplying the MLEs with probit link for ordinal data by 1.75, denoted as MLLOR*; (2) to evaluate the performance of the BLOR in the presence of outliers. We used the value 1.75 because, according to the literature review, it is the most reasonable value (Savalei 2006).

To reach these two goals, two data sets were simulated. Both simulation studies were carried out with the following liability:

$$l_{ij}=x_{i}^{T}\beta +\varepsilon_{ij},$$

where i=1,…,40 and $j=1,\ldots ,n_{i}$, $\beta^{T}=[-6,\;5,\;7]$ and the vectors $x_{i}^{T}=[x_{i1},x_{i2},\;x_{i3}]\;$ have been drawn independently, with components following a uniform distribution within the interval [−0.1, 0.1]. The threshold parameters used were γ_1_= −0.8416, γ_2_= −0.2533, γ_3_= −0.2533, and γ_4_= −0.8416. Then the response variable was generated as follows:

$$y_{ij}=\{\begin{array}{c} 1\;if\;-\infty <l_{ij}<\gamma_{1},\\ 2\;if\;\gamma_{1}<l_{ij}<\gamma_{2},\\ \vdots \\ 5\;if\;\gamma_{4}<l_{ij}<\infty \end{array}$$

For simulated data set 1, we used four values of sample size *n_i_* = 5, 10, 20, and 40 and all the $\varepsilon_{ij}$ were drawn independently from a *L*(0,1), whereas for simulated data set 2, we used only one sample size (*n_i_* = 40) and the error terms ($\varepsilon_{ij}$) were obtained from two distributions [*L*(0,1) and a student's *t* distribution with four degrees of freedom, denoted as t4]. We studied four scenarios: Scenario 1, the percentage of outliers (PO) from the t4 was 5% and the remaining percentage was obtained from the *L*(0,1) distribution; Scenario 2: the PO from the t4 was 10%, and 90% from the *L*(0,1); Scenario 3: the PO from the t4 was 20%, and 80% from the *L*(0,1); and Scenario 4: the PO from the t4 was 30%, and 70% from the *L*(0,1).

The MLE estimates for the ordinal regression were obtained using the polr function of the MASS package in R (R Core Team 2015). It is important to point out that the priors used for the Bayesian methods were not informative for $\beta |\sigma_{\beta }^{2}∼N(\beta_{0}^{T}=[0,0,0],10000\times I_{3})$, and for the hyperparameters for thresholds, we used $\gamma_{\min}=-4$ and $\gamma_{\max}=4$. We computed 20,000 Markov chain Monte Carlo (MCMC) samples. Bayes estimates were computed using 10,000 samples because the first 10,000 were discarded as burn-in.

### Model implementation

The Gibbs sampler described previously for the BLOR model was implemented with the R-software (R Core Team 2015). Implementation was done with a Bayesian approach and MCMC through the Gibbs sampler algorithm, which samples sequentially from the full conditional distributions until it reaches a stationary process, converging with the joint posterior distribution (Gelfand and Smith 1990). In the real data, to reduce the potential impact of MCMC errors on prediction accuracy, we performed a total of 60,000 iterations with a burn-in of 20,000, so that 40,000 samples were used for inference. We did not apply thinning of the chains, following the suggestions of Geyer (1992), Maceachern and Berliner (1994), and Link and Eaton (2012), who provide justification for the ban on subsampling MCMC output for approximating simple features of the target distribution (*e.g.*, means, variances and percentiles), since thinning is neither necessary nor desirable, and unthinned chains are more precise. It is important to point out that implementation of the BLOR model for the real data sets was done using the hyperparameters $\nu_{\beta }=3$, $S_{\beta }=0.001,\beta_{0}=0,\;\Sigma_{0}=10000\times I_{p},\;)$, *γ*_min_ = −1000 and *γ*_max_= −1000 for thresholds parameters, all of which were chosen to lead weakly informative priors.

### Assessing prediction accuracy

We used cross-validation to estimate the prediction accuracy of the proposed models. The data set was divided into training and validation sets 10 times, with 90% of the data set used for training and 10% for testing; this was done only for the pooled data. The training set was used to fit the model and the validation set was used to evaluate the prediction accuracy of the proposed models. Since the phenotype response is ordinal categorical, we used the Brier score (Brier 1950) to measure prediction ability, which is equal to

$$BS=n^{-1}\overset{n}{\underset{i=1}{\sum }}\overset{C}{\underset{c=1}{\sum }}{\left({\hat{\pi }}_{ic}-d_{ic}\right)}^{2}$$ (10)

where BS denotes the Brier Score, and *d_ic_* takes a value of 1 if the ordinal categorical response observed for individual *i* falls into category *c*; otherwise, *d_ic_* = 0. This scoring rule uses all the information contained in the predictive distribution, not just a small portion like the hit rate or the log-likelihood score. Therefore, it is a reasonable choice for comparing categorical regression models, although there are other scoring rules that also have good properties. The range of BS in equation (10) is between 0 and 2. For this reason, we divided Brier scores (BS)/2 to get the BS bounded between 0 and 1; lower scores imply better predictions. It is important to point out that we also used the BS when analyzing the full data sets. We also used the deviance information criterion (DIC) to compare Bayesian models, as suggested by Gelman *et al.* (2003); here, the lower the DIC, the better the model.

### Data availability

The two real data sets and two simulated data sets together with R codes are deposited in the link http://hdl.handle.net/11529/10254. The phenotypic data for GLS in three environments (México, Zimbabwe, and Colombia) for the 278 maize lines, the 46,347 SNPs, and the R scripts developed to fit the predictive models used in this study are given in the files PhenoGLS.RData, and MarkersGLSFinal.RDat. The repository also contains the Septoria genotypic and phenotypic data sets, in files SeptoriaGenotypic.RDat, and SeptoriaPhenotypic. RDat, respectively. The R codes to generate the simulated data sets 1 and 2, and for analyzing the real data set from Colombia are directly given in Appendices B, C, and D, respectively. 

## Results

In the following sections, we investigate the performance of the proposed BLOR estimator through a simulation study and with real data.

### Simulated data set

In Table 1, we report average estimates obtained by all methods, along with SDs; all the results are based on 50 replications. From Table 1, it is clear that as the sample size increases, the average biases and SD decrease in all cases. This confirmed the consistency properties of all the estimates. Table 1 also shows that, in general, the point estimates of the Bayesian estimates (BLOR and BLOR*) are similar to the MLEs (MLLOR, and MLLOR*, which was approximated with the MLE with probit ordinal regression); however, the approximations (BLOR* and MLLOR*) have greater bias and SD. BLOR has less bias and SD in most of the studied parameters, producing better parameter estimates than the MLLOR (which is the correct method that use maximum likelihood with the logit link function). For this reason, the proposed BLOR is an excellent alternative. However, a more in-depth simulation study is required to ensure that these findings are valid for all possible scenarios.

**Table 1:** Simulated data set 1: Average values (Mean) and SD of MLEs and the Bayesian estimators, with four sample sizes (*n_i_*)

*n_i_*	Parameter	True Value	BLOR		BLOR*		MLLOR		MLLOR*	
			Mean	SD	Mean	SD	Mean	SD	Mean	SD
	$\beta_{1}$	−6	**-6.141**	1.935	−6.711	2.117	−6.380	2.489	−6.826	2.668
	$\beta_{2}$	−5	**-4.957**	2.262	−5.546	2.726	−5.596	2.731	−5.927	2.872
	$\beta_{3}$	7	7.550	2.746	7.815	3.112	6.659	2.698	**7.110**	2.885
5	$\gamma_{1}$	−0.842	**-0.851**	0.190	−0.937	0.152	−0.883	0.177	−0.942	0.185
	$\gamma_{2}$	−0.253	**-0.254**	0.154	−0.271	0.142	−0.277	0.167	−0.298	0.179
	$\gamma_{3}$	0.253	0.274	0.170	0.328	0.171	**0.262**	0.203	0.281	0.218
	$\gamma_{4}$	0.842	0.878	0.171	0.967	0.151	**0.863**	0.211	0.920	0.220
	$\beta_{1}$	−6	−6.224	1.562	−6.534	1.650	**-6.118**	1.673	−6.480	1.767
	$\beta_{2}$	−5	**-4.987**	1.825	−5.433	1.901	−4.717	1.500	−5.022	1.619
	$\beta_{3}$	7	7.306	1.971	7.762	1.825	6.606	1.836	**7.038**	1.939
10	$\gamma_{1}$	−0.842	**-0.843**	0.100	−0.926	0.147	−0.847	0.127	−0.907	0.135
	$\gamma_{2}$	−0.253	**-0.239**	0.097	−0.284	0.131	−0.273	0.110	−0.296	0.119
	$\gamma_{3}$	0.253	0.276	0.113	0.272	0.123	0.233	0.110	**0.249**	0.120
	$\gamma_{4}$	0.842	0.861	0.116	0.920	0.124	**0.841**	0.115	0.897	0.123
	$\beta_{1}$	−6	−6.122	1.063	−6.278	1.390	**-6.030**	0.936	−6.422	1.017
	$\beta_{2}$	−5	**-5.099**	1.262	−5.538	1.103	−5.181	0.962	−5.488	1.019
	$\beta_{3}$	7	7.271	1.114	7.479	1.394	**7.243**	1.118	7.669	1.183
20	$\gamma_{1}$	−0.842	−0.849	0.108	−0.917	0.100	**-0.847**	0.073	−0.905	0.077
	$\gamma_{2}$	−0.253	**-0.252**	0.106	−0.291	0.094	−0.250	0.069	−0.270	0.074
	$\gamma_{3}$	0.253	0.259	0.091	0.261	0.099	**0.254**	0.068	0.272	0.073
	$\gamma_{4}$	0.842	0.860	0.097	0.883	0.106	**0.850**	0.079	0.907	0.084
	$\beta_{1}$	−6	**-6.058**	0.804	−6.467	0.924	−6.199	0.719	−6.563	0.783
	$\beta_{2}$	−5	−5.175	0.817	−5.231	0.879	−4.804	0.791	**-5.101**	0.856
	$\beta_{3}$	7	7.163	0.815	7.674	0.958	**7.093**	0.867	7.528	0.904
40	$\gamma_{1}$	−0.842	−0.844	0.065	−0.911	0.069	**-0.841**	0.051	−0.899	0.055
	$\gamma_{2}$	−0.253	**-0.258**	0.053	−0.278	0.064	−0.248	0.05	−0.267	0.056
	$\gamma_{3}$	0.253	0.255	0.052	0.267	0.060	**0.252**	0.044	0.271	0.048
	$\gamma_{4}$	0.842	0.856	0.054	0.900	0.071	**0.835**	0.047	0.893	0.050

BLOR* and MLLOR* use the parameter estimates of BPOR and MLLOR and approximate BLOR and MLLOR with $logit\left(u\right)=1.75\times {\text{Φ}}^{-1}(u)$. The best model has the value for the parameter closer to the true value; these are presented in bold. MLEs, maximum likelihood estimators; BLOR, Bayesian logistic ordinal regression; MLLOR, maximum likelihood logistic ordinal regression.

Table 2 shows that the smaller the PO, the less bias there is in the parameter estimates under the four methods (two Bayesians and two under Maximum Likelihood). However, under the two Bayesian methods, the proposed BLOR showed less bias than BLOR*, and this behavior is observed for all parameters and PO under study, except when the PO is 30% and for the parameter *γ*_2_. Under the two maximum likelihood methods, the approximate method (MLLOR*) showed greater bias in all scenarios and parameters. Finally, although BLOR and MLLOR produced better results in term of bias, in most cases, MLLOR was better than BLOR.

**Table 2:** Simulated data set 2: average values (Mean) and SD of MLEs and Bayesian estimators, with four POs

PO	Parameter	True Value	BLOR		BLOR*		MLLOR		MLLOR*	
			Mean	SD	Mean	SD	Mean	SD	Mean	SD
	$\beta_{1}$	−6	**-5.994**	0.824	−6.525	0.915	−6.230	0.723	−6.608	0.766
	$\beta_{2}$	−5	−5.088	0.880	−5.678	0.864	**-5.015**	0.763	−5.324	0.815
	$\beta_{3}$	7	7.183	0.802	7.607	0.893	**7.111**	0.651	7.535	0.689
5	$\gamma_{1}$	−0.842	−0.862	0.066	−0.923	0.067	**-0.853**	0.050	−0.910	0.053
	$\gamma_{2}$	−0.253	−0.263	0.071	−0.281	0.059	**-0.256**	0.048	−0.276	0.051
	$\gamma_{3}$	0.253	**0.258**	0.068	0.285	0.051	0.261	0.040	0.281	0.043
	$\gamma_{4}$	0.842	0.861	0.067	0.928	0.058	**0.856**	0.041	0.914	0.043
	$\beta_{1}$	−6	**-**6.156	0.854	−6.670	0.979	**-6.136**	0.607	−6.504	0.647
	$\beta_{2}$	−5	−5.359	0.899	−5.544	1.094	**-5.149**	0.610	−5.459	0.639
	$\beta_{3}$	7	7.349	0.830	7.739	0.856	**7.109**	0.645	7.536	0.682
10	$\gamma_{1}$	−0.842	−0.883	0.063	−0.925	0.076	**-0.866**	0.048	−0.924	0.050
	$\gamma_{2}$	−0.253	−0.266	0.070	−0.267	0.072	**-0.265**	0.052	−0.285	0.056
	$\gamma_{3}$	0.253	**0.260**	0.071	0.313	0.065	0.264	0.051	0.284	0.055
	$\gamma_{4}$	0.842	0.868	0.068	0.964	0.082	**0.861**	0.052	0.918	0.055
	$\beta_{1}$	−6	−6.529	0.730	−6.828	0.903	**-6.345**	0.645	−6.709	0.689
	$\beta_{2}$	−5	**-5.244**	0.759	−5.722	0.877	−5.275	0.629	−5.589	0.671
	$\beta_{3}$	7	7.525	0.784	7.860	0.758	**7.344**	0.735	7.780	0.772
20	$\gamma_{1}$	−0.842	−0.915	0.065	−0.972	0.060	**-0.883**	0.049	−0.942	0.053
	$\gamma_{2}$	−0.253	−0.275	0.059	−0.295	0.057	**-0.260**	0.044	−0.280	0.048
	$\gamma_{3}$	0.253	0.279	0.063	0.297	0.059	**0.271**	0.047	0.291	0.051
	$\gamma_{4}$	0.842	0.922	0.060	0.977	0.060	**0.895**	0.053	0.954	0.057
	$\beta_{1}$	−6	−6.794	0.803	−7.011	1.013	**-6.555**	0.654	−6.916	0.691
	$\beta_{2}$	−5	−5.652	0.754	−5.827	0.754	**-5.298**	0.634	−5.590	0.666
	$\beta_{3}$	7	8.065	0.894	8.351	0.881	**7.491**	0.752	7.925	0.798
30	$\gamma_{1}$	−0.842	−0.972	0.071	−1.004	0.075	**-0.898**	0.041	−0.956	0.044
	$\gamma_{2}$	−0.253	−0.301	0.060	−0.296	0.072	**-0.269**	0.044	−0.289	0.047
	$\gamma_{3}$	0.253	**0.279**	0.067	0.318	0.069	0.286	0.044	0.306	0.047
	$\gamma_{4}$	0.842	0.944	0.068	1.023	0.060	**0.922**	0.044	0.981	0.046

The outliers were generated with a student's *t* distribution with four degrees of freedom. BLOR* and MLLOR* use the parameter estimates of BPOR and MLLOR and approximate BLOR and MLLOR with $logit\left(u\right)=1.75\times {\text{Φ}}^{-1}(u)$. The best model has the value for the parameter closer to the true value; these are presented in bold. MLEs, maximum likelihood estimators; POs, percentages of outliers; BLOR, Bayesian logistic ordinal regression; MLLOR, maximum likelihood logistic ordinal regression.

### Real data sets

Next we compared BLOR and BPOR with the real data set (GLS and Septoria data) described in the *Materials and Methods*. However, because with this data set the number of parameters (betas and thresholds) to be estimated is high, we compared both models with point and interval estimates of the probabilities estimated for each category on the four- and five-point ordinal scale for each data set studied. In Table 3, we compare BLOR, BPOR, and BLOR* (this is the approximate method since the estimates resulting from BPOR were used to approximate BLOR with $\log it\left(u\right)=1.75\times {\text{Φ}}^{-1}(u))$ with five data sets (three locations, the resulting pooling of the three locations and the Septoria data set) made up from the data sets described in the section *Materials and Methods*. First, when comparing BLOR with BLOR*, we see that there are differences in the point probability estimates, and the lower the probabilities, the greater the differences. However, in general, the widths of the credible sets are similar. Second, when comparing BLOR with BPOR, we see that the point and credible sets for each location and Septoria produced similar results; however, for the pooled data, BLOR produced estimates with narrower confidence sets than BPOR (Table 3). We observed that the estimates produced using BPOR are less accurate because wider confidence sets are produced when the data are pooled; this could be because the assumption of errors normally distributed with mean zero and variance one when the data are pooled is not fulfilled. Also, there were no differences in the BS of the three models (BLOR, BLOR*, and BPOR).

Comparing the models with DIC, we see that in Zimbabwe, BPOR produced the lowest DIC (DIC = 4150.18), whereas BLOR* produced the highest (DIC = 4156.86). However, the DIC of BLOR (DIC = 4150.29) was very close to that of BPOR. In México, the lowest DIC was for BLOR (DIC = 1313.82), whereas the highest was for BLOR* (DIC = 1315.21). In Colombia, BLOR had the lowest DIC (2577.92) and BLOR* had the highest (2581.66). In the Septoria data set, BPOR had the lowest DIC (651.79) and BLOR had the highest (654.33). Finally, in the pooled data, BLOR (8327.36) also had the lowest DIC, whereas BLOR* had the highest (8339.539). All these results shows that even approximations that did a reasonable job (BLOR*) were sometimes very far from the exact methods (BLOR and BPOR). For this reason, whenever possible, an exact model (BLOR or BPOR) should be chosen. The fact that BPOR is sometimes the best (lower DIC) implies that for these data sets, the assumption $\varepsilon_{ij}∼N\left(0,1\right)$ in Equation (1) is enough.

Finally, in Table 4 we present the BS for the testing sets of the pooled real GLS data with 10 cross-validations and 90% of data used for the training set and 10% for the testing set. For models BLOR and BPOR, the BS are almost identical, which means that, with regard to prediction, both models had a similar performance. However, although the approximation method (BLOR*) produced a higher BS, its prediction accuracy was very close to that of BLOR and BPOR. Although we found that the three models had a similar performance regarding prediction accuracy with this data set, more in-depth research is required to validate this observation.

**Table 3:** Real data sets: GLS and Septoria data sets

Model	Set (DIC)	Statistic	Probability of Each Category					BS
			1	2	3	4	5	
		Mean	0.025	0.256	0.392	0.196	0.132	0.363
	Zimbabwe	L	0.018	0.236	0.368	0.173	0.112	0.363
	(4150.29)	U	0.034	0.278	0.415	0.216	0.149	0.363
		Mean	0.054	0.442	0.267	0.169	0.069	0.350
BLOR	México	L	0.035	0.399	0.228	0.139	0.050	0.349
	(1313.82)	U	0.075	0.480	0.308	0.200	0.092	0.352
		Mean	0.204	0.248	0.259	0.212	0.077	0.390
	Colombia	L	0.179	0.219	0.232	0.187	0.058	0.389
	(2577.92)	U	0.233	0.279	0.287	0.239	0.095	0.391
		Mean	0.083	0.284	0.332	0.198	0.104	0.377
	Pooled	L	0.069	0.269	0.315	0.184	0.093	0.377
	(8327.36)	U	0.093	0.299	0.350	0.211	0.119	0.377
		Mean	0.077	0.150	0.432	0.341	−	0.335
	Septoria	L	0.049	0.112	0.376	0.294	−	0.333
	(654.33)	U	0.110	0.195	0.493	0.388	−	0.338
		Mean	0.032	0.237	0.416	0.190	0.124	0.363
	Zimbabwe	L	0.025	0.219	0.389	0.171	0.110	0.363
	(4156.86)	U	0.039	0.258	0.443	0.211	0.140	0.365
		Mean	0.057	0.436	0.280	0.156	0.070	0.350
BLOR*	México	L	0.041	0.392	0.242	0.128	0.054	0.350
	(1315.21)	U	0.075	0.479	0.323	0.187	0.089	0.353
		Mean	0.193	0.260	0.279	0.194	0.074	0.390
	Colombia	L	0.168	0.226	0.248	0.168	0.060	0.389
	(2581.66)	U	0.220	0.292	0.310	0.223	0.089	0.392
		Mean	0.082	0.277	0.358	0.184	0.100	0.377
	Pooled	L	0.074	0.259	0.341	0.168	0.090	0.377
	(8339.54)	U	0.091	0.294	0.375	0.200	0.109	0.378
		Mean	0.075	0.137	0.457	0.332	−	0.334
	Septoria	L	0.051	0.098	0.392	0.268	−	0.330
	(652.50)	U	0.104	0.176	0.527	0.393	−	0.341
		Mean	0.025	0.253	0.392	0.199	0.132	0.363
	Zimbabwe	L	0.017	0.233	0.366	0.179	0.113	0.363
	(4150.18)	U	0.033	0.274	0.416	0.219	0.148	0.363
		Mean	0.054	0.440	0.265	0.171	0.070	0.350
BPOR	México	L	0.036	0.399	0.229	0.142	0.051	0.350
	(1314.70)	U	0.076	0.479	0.304	0.203	0.092	0.352
		Mean	0.206	0.249	0.261	0.209	0.075	0.390
	Colombia	L	0.176	0.221	0.230	0.183	0.058	0.389
	(2578.42)	U	0.233	0.277	0.293	0.233	0.095	0.391
		Mean	0.071	0.256	0.331	0.218	0.123	0.377
	Pooled	L	0.042	0.183	0.313	0.191	0.104	0.374
	(8329.84)	U	0.086	0.287	0.350	0.286	0.171	0.389
		Mean	0.075	0.150	0.430	0.345	−	0.334
	Septoria	L	0.047	0.110	0.369	0.282	−	0.330
	(651.79)	U	0.109	0.191	0.500	0.402	−	0.339

BLOR* use the parameter estimates of the BPOR and approximate the BLOR with $logit\left(u\right)=1.75\times {\text{Φ}}^{-1}(u)$. Point probability estimates, credible sets for each category, DIC, and BS for the threshold Bayesian ridge regression. L and U denote lower and upper confidence sets, respectively. GLS, Gray leaf spot; DIC, deviance information criterion; BS, Brier scores; BLOR, Bayesian logistic ordinal regression; BPOR, Bayesian probit ordinal regression.

**Table 4:** GLS data set

Model	Brier Scores		
	Mean	Min	Max
BLOR	0.373	0.365	0.381
BLOR*	0.374	0.364	0.382
BPOR	0.373	0.365	0.381

BLOR* uses the parameter estimates of BPOR and approximates BLOR with $logit\left(u\right)=1.75\times {\text{Φ}}^{-1}(u)$. Brier scores (mean, minimum and maximum; lower scores indicate better prediction) evaluated for validation samples from the pooled data. GLS, Gray leaf spot; BLOR, Bayesian logistic ordinal regression; BPOR, Bayesian probit ordinal regression.

This paper proposes a method for BLOR using the Pólya-Gamma data augmentation approach of Scott and Pillow (2013), which produces a Gibbs sampler with full conditional distributions similar to that of the BPOR model of Albert and Chib (1993). The proposed method is reduced to the BPOR model when the sampled values, $\omega_{ij}$, from the Pólya-Gamma distribution in Equation (4) are set to 1. This is an advantage because with the proposed model, researchers can perform an exact logistic or probit ordinal regression without having to do approximations to perform a logistic ordinal regression. The performance of the proposed method was compared with the approximation using the probit ordinal regression model in a small simulation study and real data sets (GLS and Septoria data sets) using a four- and five-point ordinal scale. On the basis of the simulation study, it is clear that the estimation of parameters using the approximation $logit\left(u\right)=(1.75){\text{Φ}}^{-1}(u)$ produces a considerable amount of bias and can give rise to wrong conclusions in association studies. However, we observed with the real data that, in terms of prediction ability, both models (BLOR and BPOR) have a similar performance even though we observed BLOR had lower DIC values in México, Colombia, and the pooled data. This means that when violation of the assumption $\varepsilon_{ij}∼N\left(0,1\right)$ in Equation (1) is not strong, any model can be used. For this reason, we observed greater accuracy (narrow confidence sets) for the BLOR model compared with the exact BPOR model (BPOR) only with the pooled data set without a covariate for location.

Although with the real data we did not observe an advantage in prediction accuracy with the proposed BLOR model, it is very well documented in statistical literature that logistic ordinal regression is more robust for dealing with outlying data, because logistic distribution has heavier tails which was corroborated in terms of parameter estimates with the simulation done using simulated data set 2. For this reason, the proposed BLOR should be preferred because it is usually not practical to test if the error term in Equation (1) is $\varepsilon_{ij}∼N\left(0,1\right)$ or L(0,1). In addition to being more robust, the proposed method also provides regression coefficients that are more interpretable because of their connection to odds ratios (Zucknick and Richardson 2014). However, this advantage does not make sense when *p >> n*, because the main driving force in Bayesian models in the case of *p >> n* is the prior and not the data (Gianola 2013). Even with this restriction, this paper unifies logistic and probit ordinal regression under a Bayesian framework and is a useful alternative for genomic-enabled prediction of ordinal categorical trials where available data sets have a larger number of parameters to estimate than observations. Also, the proposed method should be preferred over BPOR when outliers are present and not easily detected. This is especially true for multidimensional data, since many times a few outliers in a data set can have strong influence on parameter estimation and inference.

Finally, it is important to point out that, to devise the method proposed in this paper, we generalized the work of Scott and Pillow (2013) for ordered categorical responses. Our method is elegant, easy to implement, and produces a unified Gibbs sampler framework useful for both the logit and the probit link. For this reason, we believe it is an appealing alternative for plant and animal researchers. Also, the proposed BLOR model can be easily extended to take into account genotype × environment interactions, which play an extremely important role in plant breeding, especially when selecting candidate genotypes.

## Appendix A

### Derivation of full conditional distributions

#### Liabilities and Pólya-Gamma values

The fully conditional posterior distribution of liability $l_{ij}$ is

P(l|ELSE)∝P(l|β,b)P(y|l,γ )

$$\propto \overset{I}{\underset{i=1}{\prod }}\overset{J}{\underset{j=1}{\prod }}f(l_{ij})\overset{C}{\underset{c=1}{\sum }}I(y_{ij}=c)I\left(\gamma_{c-1}<l_{ij}<\gamma_{c}\right)$$

$$\propto \overset{I}{\underset{i=1}{\prod }}\overset{J}{\underset{j=1}{\prod }}\frac{\exp\left(-l_{ij}+x_{ij}^{T}\beta +b_{i}\right)}{{\left[1+\exp\left(-l_{ij}+x_{ij}^{T}\beta +b_{i}\right)\right]}^{2}}\overset{C}{\underset{c=1}{\sum }}I(y_{ij}=c)I\left(\gamma_{c-1}<l_{ij}<\gamma_{c}\right)$$

$$\propto \overset{I}{\underset{i=1}{\prod }}\overset{J}{\underset{j=1}{\prod }}2^{-2}\overset{\infty }{\underset{0}{\int }}\exp\left[-\frac{\omega_{ij}{\left(-l_{ij}+x_{\mathrm{ij}}^{T}\beta +b_{i}\right)}^{2}}{2}\right]P(\omega_{ij};b=2,d=0)d\omega_{ij}\overset{C}{\underset{c=1}{\sum }}I(y_{ij}=c)I\left(\gamma_{c-1}<l_{ij}<\gamma_{c}\right)$$

The last inequality was obtained using a technique called the Pólya-Gamma method (Scott and Pillow 2013), which is useful when working with logistic likelihoods, and has the form

$$\frac{{\left(e^{\psi }\right)}^{a}}{{\left(1+e^{\psi }\right)}^{b}}=2^{-b}e^{\kappa \psi }\overset{\infty }{\underset{0}{\int }}e^{-\frac{\omega \psi^{2}}{2}}P(\omega ;b,0)d\omega$$

where κ=a−b/2 and P(ω;b,d=0) denotes the density of the random variable ω∼PG(b,d=0), where PG(b,d) denotes a Pólya-Gamma distribution with parameters *b* and *d* and density

$$P\left(\omega ;b,d\right)=\left\{cosh^{b}\left(\frac{d}{2}\right)\right\}\;\;\frac{2^{b-1}}{\text{Γ}(b)}\overset{\infty }{\underset{k=0}{\sum }}{\left(-1\right)}^{n}\frac{\text{Γ}(n+b)(2n+b)}{\text{Γ}(n+1)\sqrt{2\pi \omega^{3}}}\text{exp}(-\frac{{\left(2n+b\right)}^{2}}{8\omega }-\frac{d^{2}}{2}\omega )$$,

where cosh denotes the hyperbolic cosine.

Then the joint distribution of $l_{ij}\;$and $\omega_{ij}$ is equal to

$$P\left(l,\omega |ELSE\right)\propto \overset{I}{\underset{i=1}{\prod }}\overset{J}{\underset{j=1}{\prod }}2^{-2}\exp\left[-\frac{\omega_{ij}{\left(-l_{ij}+x_{ij}^{T}\beta +b_{i}\right)}^{2}}{2}\right]P(\omega_{ij};2,0)\overset{C}{\underset{c=1}{\sum }}I(y_{ij}=c)I\left(\gamma_{c-1}<l_{ij}<\gamma_{c}\right)$$

Therefore, the fully conditional posterior distribution of liability $l_{ij}$ is a truncated normal distribution and its density is

$$P\left(l_{ij}|ELSE\right)=\frac{\varphi (x_{\mathrm{ij}}^{T}\beta +b_{i},1/\sqrt{\omega_{ij}})}{\text{Φ}\left(\gamma_{c}-x_{ij}^{T}\beta -b_{i}\right)-\text{Φ}\left(\gamma_{c-1}-x_{ij}^{T}\beta -b_{i}\right)}$$

where φ(.) is a normal density with parameters as indicated in the argument, Φ is the cumulative distribution function of a normal density with mean $x_{ij}^{T}\beta +b_{i}\;$and variance **$1/\sqrt{\omega_{ij}}$**, and the fully conditional posterior distribution of $\omega_{ij}$ is

$$P\left(\omega_{ij}|ELSE\right)\propto 2^{-2}\exp\left[-\frac{\omega_{ij}{\left(-l_{ij}+x_{ij}^{T}\beta +b_{i}\right)}^{2}}{2}\right]P(\omega_{ij};2,0)\propto \exp\left[-\frac{\omega_{ij}{\left(-l_{ij}+x_{ij}^{T}\beta +b_{i}\right)}^{2}}{2}\right]P(\omega_{ij};2,0)$$

and from here and equation (5) of Polson *et al.* (2013) we get that

$$\omega_{ij}|ELSE∼PG(2,-l_{ij}+x_{ij}^{T}\beta +b_{i})$$

#### Regression coefficients (*β*)

First note that the fully conditional posterior of l, β,ω is

$$P\left(l,\beta ,\omega |ELSE\right)\propto P\left(l|\;\beta ,\;b\right)P\left(y|l,\gamma \;\right)P(\omega )P(\beta |\sigma_{\beta }^{2})$$

$$\propto \exp\left(-\frac{1}{2}{\left(-l+X\beta +Zb\right)}^{T}D_{\omega }\left(-l+X\beta +Zb\right)\right)P(\omega )P(\beta |\sigma_{\beta }^{2})$$

where $P\left(\omega \right)=\overset{I}{\underset{i=1}{\prod }}\overset{m_{i}}{\underset{j=1}{\prod }}P\left(\omega_{ij};2,0\right)$. Then, the fully conditional posterior distribution of ***β*** is

$$P\left(\beta |ELSE\right)\propto \exp\left(-\frac{1}{2}{\left(-l+X\beta +Zb\right)}^{T}D_{\omega }\left(-l+X\beta +Zb\right)-\frac{1}{2}{\left(\beta -\beta_{0}\right)}^{T}(\Sigma_{0}^{-1}\sigma_{\beta }^{-2})(\beta -\beta_{0})\right)$$

$$\propto \exp\left(-\frac{1}{2}[\beta^{T}\left(\Sigma_{0}^{-1}\sigma_{\beta }^{-2}+X^{T}D_{\omega }X\right)\beta -2{\left(\Sigma_{0}^{-1}\sigma_{\beta }^{-2}\beta_{0}-X^{T}D_{\omega }Zb+X^{T}D_{\omega }l\right)}^{T}\beta ]\right)$$

$$\propto \exp\left(-\frac{1}{2}[{\left(\beta -{\tilde{\beta }}_{0}\right)}^{T}{\tilde{\Sigma }}_{0}^{-1}(\beta -{\tilde{\beta }}_{0})]\right)$$

where ${\tilde{\Sigma }}_{0}={\left(\Sigma_{0}^{-1}\sigma_{\beta }^{-2}+X^{T}D_{\omega }X\right)}^{-1}$, ${\tilde{\beta }}_{0}={\tilde{\Sigma }}_{0}(\Sigma_{0}^{-1}\sigma_{\beta }^{-2}\beta_{0}-X^{T}D_{\omega }Zb+X^{T}D_{\omega }l)$. It is important to point out that if we use a prior for β∝Constant (improper uniform distribution), then in ${\tilde{\Sigma }}_{0}$ and ${\tilde{\beta }}_{0}$ we need to make **0** the term $\Sigma_{0}^{-1}\sigma_{\beta }^{-2}$. Finally, the fully conditional posterior of ***β*** is

$$\beta |ELSE∼N_{p}({\tilde{\beta }}_{0},{\tilde{\Sigma }}_{0})$$

#### Polygenic effects (b)

Now the fully conditional posterior of ***b*** is given as

$$P\left(b|ELSE\right)\propto \exp\left(-\frac{1}{2}{\left(-l+X\beta +Zb\right)}^{T}D_{\omega }\left(-l+X\beta +Zb\right)\right)\;P(b|\sigma_{b}^{2})$$

$$\propto \exp\left\{-\frac{1}{2}\;\left[b^{T}\left(\sigma_{b}^{-2}G^{-1}+Z^{T}D_{\omega }Z\right)b-2\;{\left(Z^{T}D_{\omega }l-Z^{T}D_{\omega }X\beta \right)}^{T}\;b\right]\right\}$$

$$\propto \exp\left\{-\frac{1}{2}\;{\left(b-\tilde{b}\right)}^{T}F^{-1}(b-\tilde{b})\right\}$$

This implies that the fully conditional posterior of ***b*** is

$$\;\;\;\;\;b|ELSE∼N_{I}\left(\tilde{b}=F\left(Z^{T}D_{\omega }l-Z^{T}D_{\omega }X\beta \right),F={\left(\sigma_{b}^{-2}G^{-1}+Z^{T}D_{\omega }Z\right)}^{-1}\right)\;$$

#### Variance of polygenic effects

Next, the conditional distribution of $\sigma_{b}^{2}$ is obtained. If $\sigma_{b}^{2}∼\;\chi^{-2}(\nu_{b},S_{b})\left(shape\;and\;scale\right)$, then

$$P\left(\sigma_{b}^{2}|ELSE\right)\propto \frac{1}{{\left(\sigma_{b}^{2}\right)}^{\frac{\nu_{b}+I}{2}+1}}\exp\left(-\frac{b^{T}G^{-1}b+\nu_{b}S_{b}}{2\sigma_{b}^{2}}\right)$$

This is the kernel of the scaled inverted $\chi^{2}$ distribution; therefore, the fully conditional posterior is

$$\sigma_{b}^{2}|ELSE∼\chi^{-2}({\tilde{\nu }}_{b}=\nu_{b}+I,{\tilde{S}}_{b}=(b^{T}G^{-1}b+\nu_{b}S_{b})/\nu_{b}+I)$$

#### Threshold effects (*γ*)

The density of the fully conditional posterior distribution of the *c*^th^ threshold, *γ_c_*, is

P(γ|ELSE)∝P(y|l,γ)P(γ)

$$\propto \overset{I}{\underset{i=1}{\prod }}\overset{J}{\underset{j=1}{\prod }}\overset{C}{\underset{c=1}{\sum }}I(y_{ij}=c)I\left(\gamma_{c-1}<l_{ij}<\gamma_{c}\right)I\left(\gamma \in T\right)$$ (A.1)

If Equation (A.1) is seen as a function of *γ_c_*, it is evident that the value of *γ_c_* must be larger than all the $l_{ij}|y_{ij}=c$ and smaller than all the $l_{ij}|y_{ij}=c+1$. Hence, as a function of *γ_c_*, Equation (A.1) leads to the uniform density

$$P\left(\gamma_{c}|ELSE\right)=\frac{1}{\min\left(l_{ij}|y_{ij}=c+1\right)-\max\left(l_{ij}|y_{ij}=c\right)}I\left(\gamma \in T\right)$$ (A.2)

Equation (A.2) corresponds to a uniform distribution on the interval $\left[\min\left\{\min\;\left(l_{ij}|y_{ij}=c+1\right),\gamma_{c+1},\gamma_{\max}\right\},\max\{\max\left(l_{ij}|y_{ij}=c\right),\gamma_{c-1},\gamma_{\min}\}\right]$ (Albert and Chib 1993; Sorensen *et al.* 1995).

#### Variance of location effects

If we give *$\sigma_{\beta }^{2}∼\;\chi^{-2}(\nu_{\beta },S_{\beta })\left(shape\;and\;scale\right)$*, then

$$P\left(\sigma_{\beta }^{2}|ELSE\right)\propto P\left(\sigma_{\beta }^{2}\right)P\left(\beta |\;\sigma_{\beta }^{2}\right)=\frac{1}{{\left(\sigma_{\beta }^{2}\right)}^{\frac{\nu_{\beta }}{2}+1}}\exp\left(-\frac{\nu_{\beta }S_{\beta }}{2\sigma_{\beta }^{2}}\right)P\left(\beta |\;\sigma_{\beta }^{2}\right)$$

$$\propto \frac{1}{{\left(\sigma_{\beta }^{2}\right)}^{\frac{\nu_{\beta }+p}{2}+1}}\exp\left(-\frac{{\left(\beta -\beta_{0}\right)}^{T}\Sigma_{0}^{-1}(\beta -\beta_{0})+\nu_{\beta }S_{\beta }}{2\sigma_{\beta }^{2}}\right)$$

This is the kernel of the scaled inverted $\chi^{2}$ distribution; therefore, the fully conditional posterior is

$$\sigma_{\beta }^{2}|ELSE∼\chi^{-2}({\tilde{\nu }}_{\beta }=\nu_{\beta }+p,{\tilde{S}}_{\beta }=[{\left(\beta -\beta_{0}\right)}^{T}{\text{Σ}}_{0}^{-1}(\beta -\beta_{0})+\nu_{\beta }S_{\beta }]/\nu_{\beta }+p)$$

## Appendix B

### R code for simulating data set 1

We used this code for each sample size studied, and thus we only need to change ni, that denotes *n*_*i*_, for the other values (10, 20, 40). For each sample size, we used this code 50 times for estimating the results in Table 1.

thetav = c(-5,-10,15)

Datos<-numeric(0)

nC=40;ni=5 ####Change this ni for other sample sizes

for(i in 1:nC)

{xi1<-runif(length(thetav),-0.1,0.1)

Eta=t(xi1)%*%thetav

for(j in 1:ni)

{L=Eta + rlogis(1, location = 0, scale = 1)

y=ifelse(L<=-0.8416,1,ifelse(L<=-0.2533,2,ifelse(L<0.2533,3,ifelse(L<0.8416,4,5))))

Datos<-rbind(Datos,c(j,y,t(xi1)))

}}

colnames(Datos) = c(’j’,’y’,’x1’,’x2’,’x3′)

Datos

## Appendix C

### R codes for simulating data set 2

We used this code for each percentage of outliers (PO) studied; for this reason, we only need to change sam.out for values: 4, 8, and 12, which represent 10, 20, and 30% of outliers. For each sample size, we used this code 50 times for estimating the results in Table 2.

thetav = c(-5,-10,15)

Datos<-numeric(0)

nC=40; ni=40, sam.out =2 ###(this 2 represents 5% of outliers)

for(i in 1:nC)

{xi1<-runif(length(thetav),-0.1,0.1)

Eta=t(xi1)%*%thetav

for(j in 1:ni)

{ L=Eta + ifelse(j>sam.out,rlogis(1, location = 0, scale = 1), rt(1, df=4, ncp=0))

y=ifelse(L<=-0.8416,1,ifelse(L<=-0.2533,2,ifelse(L<0.2533,3,ifelse(L<0.8416,4,5))))

Datos<-rbind(Datos,c(j,y,t(xi1)))

}}

colnames(Datos) = c(’j’,’y’,’x1’,’x2’,’x3′)

Datos

## Appendix D

### R code for fitting the proposed BLOR for Colombia

This code was used for all the analysis given in Table 3.

#####Code for the Bayesian Ordinal REgression ##################################

rm(list=ls()) # remove everything from memory in the working environment.

library(matrixcalc)

library(BayesLogit)

library(mvtnorm)

##### We load the matrix of markers ############################################

load(’MarkersFinal.RData’)

M=as.matrix(X)

DataOrd1=data.frame(read.table(’GLSdataOsval1.csv’,sep=’,’,h=T));

DataOrd=na.omit(DataOrd1)

DataOrd=DataOrd[order(DataOrd$Stock), ]

DataOrd=subset(DataOrd, Loc==’Colombia’)

y=DataOrd[,4]

XD=DataOrd[,1:4]

##### Calculating the marker-derived genomic relationship matrix (GRM) #########

M<-scale(M,center=TRUE,scale=TRUE)

G<-tcrossprod(M)/ncol(M)

LL=t(chol(G))

##### Incidence matrix and covariance for main eff. of lines ###################

XD$Stock<-factor(x=XD$Stock, levels=rownames(M), ordered=TRUE)

ZL<-model.matrix(∼XD$Stock-1)

Z=(ZL%*%LL)

X=Z

tX=t(X)

##### Starting values of Beta0, gammas(thresholds) #############################

gammas<-c(-Inf,-3.11,-2.54,-1.89,-1.27,Inf)

nt=length(gammas)-2

m = dim(X)[1]

nB=dim(X)[2]

In =diag(nB)

p=nB

Betas=rep(0,,dim(X)[2])

SigmaB=1

L<-rep(0,m)

##### Priors for the parameters ################################################

vB=3

SB=0.001

gam.Min=-1000

gam.Max=1000

##### Number of iterations requiered for the Gibbs sampler #####################

Niter<-60000

#### Matrices for saving the output ############################################

MBetas<-matrix(nrow=Niter,ncol=nB)

Mgammas<-matrix(nrow=Niter,ncol=nt)

Mvar_Beta<-matrix(nrow=Niter,ncol=1)

#### Function for sampling normal truncated values###############################

ntruncada<-function(lo,hi,media,std)

{

U = runif(m)

F0 = pnorm(lo,media,std)

c = pnorm(hi,media,std)-F0

muestra = qnorm(c*U+F0)*std+media

}

##### Gibbs sampler ############################################################

for(i in 1:Niter)

{

##### Linear predictor #########################################################

eta=as.numeric(Betas)

##### Samples from polya gamma distribution ####################################

##### Replacing w=rpg(num=m,2,-L+eta) with w=rep(1,m) we get the BPOR #######

w=rpg(num=m,2,-L+eta)

D=diag(w)

##### Sample liabilities (L) from a truncated normal distribution ##############

L = ntruncada(gammas[y],gammas[y+1],media=eta,1/sqrt(w))

##### Sample of thresholds #####################################################

newgammas<-numeric(0)

for(k in 2:(nt+1))

{

lo<-max(c(max(L[y==k-1]),gammas[k-1],gam.Min))

hi<-min(c(min(L[y==k]),gammas[k+1],gam.Max))

newgammas<-append(newgammas,runif(1,lo,hi))

}

gammas<-c(-Inf,newgammas,Inf)

##### Sample of Betas ##########################################################

C = 1/SigmaB*In

X_tD = tX%*%D

MM=X_tD%*%X

diag(MM)=diag(MM)+diag(C)

S_a=solve(MM,tol=1e-19)

mu_a = S_a%*%(t(X)%*%D%*%L)

Betas=t(rmvnorm(1, mean =mu_a, sigma =S_a))

##### Sample of sigma_Beta##########################################################

SigmaB = 1/rgamma(1,shape=(vB+p)/2,(SB+tcrossprod(Betas))/2)

##### Saving output ############################################################

MBetas[i,]=t(Betas)

Mgammas[i,]=t(newgammas)

Mvar_Beta[i]=SigmaB

##### Printing some posterior values estimated #################################

cat(’Niter’,i,’SigmaB =’,SigmaB,’Beta1=’,Betas[1],’\n’)}
